# Novel in silico screening system for plant defense activators using deep learning-based prediction of reactive oxygen species accumulation

**DOI:** 10.1186/s13007-023-01118-7

**Published:** 2023-12-08

**Authors:** Masayuki Kogoshi, Daiki Nishio, Nobutaka Kitahata, Hayato Ohwada, Kazuyuki Kuchitsu, Hideyuki Mizuno, Takamitsu Kurusu

**Affiliations:** 1 Department of Engineering and Management, Suwa University of Science, 5000-1 Toyohira, Chino, Nagano 391-0292 Japan; 2 Department of Applied Biological Science, Tokyo University of Science, 2641 Yamazaki, Noda, Chiba 278-8510 Japan; 3 Department of Industrial and Systems Engineering, Tokyo University of Science, 2641 Yamazaki, Noda, Chiba 278-8510 Japan

**Keywords:** Reactive oxygen species (ROS), Chemical property, Deep neural network (DNN), In silico screening, Pesticides, Plant defense activators

## Abstract

**Background:**

Plant defense activators offer advantages over pesticides by avoiding the emergence of drug-resistant pathogens. However, only a limited number of compounds have been reported. Reactive oxygen species (ROS) act as not only antimicrobial agents but also signaling molecules that trigger immune responses. They also affect various cellular processes, highlighting the potential ROS modulators as plant defense activators. Establishing a high-throughput screening system for ROS modulators holds great promise for identifying lead chemical compounds with novel modes of action (MoAs).

**Results:**

We established a novel in silico screening system for plant defense activators using deep learning-based predictions of ROS accumulation combined with the chemical properties of the compounds as explanatory variables. Our screening strategy comprised four phases: (1) development of a ROS inference system based on a deep neural network that combines ROS production data in plant cells and multidimensional chemical features of chemical compounds; (2) in silico extensive-scale screening of seven million commercially available compounds using the ROS inference model; (3) secondary screening by visualization of the chemical space of compounds using the generative topographic mapping; and (4) confirmation and validation of the identified compounds as potential ROS modulators within plant cells. We further characterized the effects of selected chemical compounds on plant cells using molecular biology methods, including pathogenic signal-triggered enzymatic ROS induction and programmed cell death as immune responses. Our results indicate that deep learning-based screening systems can rapidly and effectively identify potential immune signal-inducible ROS modulators with distinct chemical characteristics compared with the actual ROS measurement system in plant cells.

**Conclusions:**

We developed a model system capable of inferring a diverse range of ROS activity control agents that activate immune responses through the assimilation of chemical features of candidate pesticide compounds. By employing this system in the prescreening phase of actual ROS measurement in plant cells, we anticipate enhanced efficiency and reduced pesticide discovery costs. The *in-silico* screening methods for identifying plant ROS modulators hold the potential to facilitate the development of diverse plant defense activators with novel MoAs.

**Supplementary Information:**

The online version contains supplementary material available at 10.1186/s13007-023-01118-7.

## Background

Development of pesticides, such as herbicides, fungicides, and plant defense activators, has been drastically decreasing because conventional visual selection methods to explore useful pesticide candidates from compounds are experimentally time-consuming and expensive [1]. Additionally, in recent years, pesticide-resistant organisms have emerged worldwide, increasing the need to develop pesticides with novel modes of action (MoAs).

Reactive oxygen species (ROS; e.g., O_2_^˗•^, H_2_O_2_, OH^•^, and ^1^O_2_) are partially reduced or excited forms of atmospheric oxygen that are thought to play crucial roles in plant cells [2]. ROS play multiple beneficial roles as signaling products capable of regulating stress responses and development [3] including diverse metabolic pathways [4, 5] and gene expression [6–8]. The reduction in excessive ROS accumulation under environmental stress conditions confer tolerance to a variety of biotic and abiotic stresses [9–12]. In contrast, pathogen recognition induces ROS production by enzymes such as NADPH oxidases in both plant and animal cells [3, 13, 14]. ROS are antimicrobial products but also serve as signaling molecules that activate immune responses [15–18], indicating that ROS modulators have the potential to function as a variety of agricultural chemicals, such as plant defense activators [19–21]. As ROS have multiple physiological action points in cells [2], the ROS screening system is a typical multi-target screening system and is expected to create lead chemical compounds with novel MoAs. However, no system has been reported to predict compound-derived ROS production triggered by biotic/abiotic stress elicitation in plant cells.

Plant defense activators have advantages over pesticides, such as avoiding the appearance of drug-resistant pathogen; however, only a few such compounds have been reported [22–25]. A wide variety of chemical compounds have been screened to identify effective plant activators that may be applicable to a broad range of crops. Notably, when cultured tobacco BY-2 cells are treated with the elicitor protein cryptogein from oomycetes, immune responses, such as persistent NADPH oxidase-mediated ROS production and programmed cell death (PCD), are induced [26–28]. Recently, a monitoring system utilizing a 96-well plate and a luminometer based on the quantification of ROS production derived from the luminescence of luminol in cultured tobacco BY-2 cells was constructed [29]. ROS production in tobacco BY-2 cultured cells triggered by a pathogenic signal molecule have been shown to be useful to screen microbe that boost plant immune responses [28]. Furthermore, a screening method was developed to identify chemical compounds that regulate ROS production and trigger immune responses in plant cells [29], suggesting the value of ROS activity as an indicative factor and underscoring the necessity for extensive-scale screening in the future. However, in silico high-throughput screening system for plant defense activators using the prediction of ROS accumulation in plant cells has not been reported.

In conventional in silico screening, the search and identification of interactors targeting specific proteins (points of action) have been conducted, but their commercialization as pesticides has not been achieved. In addition, compounds exhibiting selectable phenotypes have been extensively isolated for visual selection by direct application to crops. Conversely, in the realm of deep learning, diverse applications are being pursued across various domains, such as drug discovery and virtual screening, owing to advancements in technology [30]. Recently, quantitative structure–activity relationship-based virtual screening was developed using a machine learning-based prediction model created by analyzing the relationship between chemical structures [31]. This method can be applied even if the target protein is unknown and predicts activity based on the characteristics of active unknown compounds.

In this study, we constructed a model system capable of inferring a diverse range of ROS activity control agents by learning the chemical features of candidate pesticide compounds. Our screening strategy comprised four phases: (1) development of an ROS inference system based on a deep neural network (DNN) that combines ROS production data *in planta* and multidimensional chemical features of compounds; (2) in silico screening of seven million commercially available compounds using the ROS inference model; (3) secondary screening by visualization of the chemical space using the generative topographic mapping (GTM) method; and (4) confirmation and validation of the identified compounds as ROS modulators in plant cells. We present a robust and valuable system for identifying compounds that modulate ROS production in plant cells and that function as plant defense activators. Further, the efficacy of our system as a primary screening tool for ROS modulators with plant defense activator properties is also discussed.

## Materials and methods

### Data set and construction of ROS inference model

**Fig. 1:**
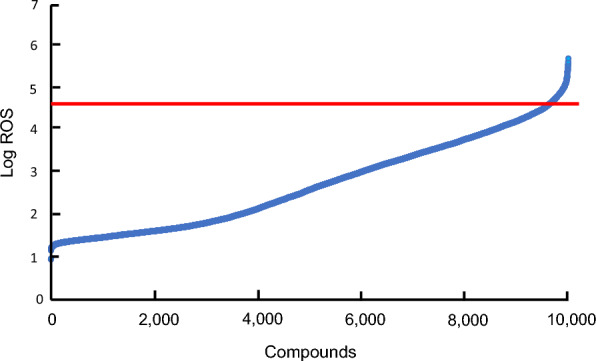
Distribution of ROS production in tobacco BY-2 cultured cells under low concentration of cryptogein-elicitor treatment. A total of 351 chemical compounds defined as ROS modulators ranked higher than the red line (log_10_[4.67]). The vertical axis represents the logarithmic conversion of ROS-dependent chemiluminescence levels in tobacco BY-2 cultured cells triggered by cryptogein (25 nM), and the horizontal axis shows the chemical compounds arranged in ascending order of their ROS-dependent chemiluminescence levels

When cultured tobacco BY-2 cells are treated with the oomycete-derived elicitor cryptogein, persistent ROS production mediated by NADPH oxidase is induced as an immune response [26, 27]. For this analysis, we used a dataset comprising chemiluminescence-dependent ROS production obtained from tobacco BY-2 cultured cells treated with 9991 chemical compounds in combination with cryptogein. A chemical library of 9991 small molecules (DIVERSet NovaCore NQ612) from MayBridge was used in this study. We constructed a library of 219-dimensional chemical features based on factors, such as the number of atoms, hydrophobicity, and other numerical values, using the structural analysis software Discovery Studio (BIOVIA). The ROS production data in tobacco BY-2 cultured cells triggered by cryptogein was subjected to logarithmic transformation (log_10_[ROS]) due to the wide range of chemiluminescence values, spanning approximately 9.5–540,000. ROS modulators were selected by converting the ROS production of each compound to a logarithmic scale and sorting the changes in ascending order. As shown in Fig. 1, 351 compounds displaying an ROS production change of ≥ log_10_[4.67] that showed a sharp change in activity among all compounds were identified as ROS modulators. The ROS production data used in this study are presented in Supplementary Information (Additional file 1).

**Fig. 2:**
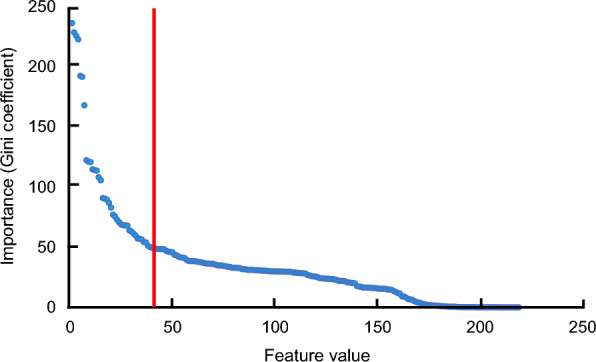
Importance of each chemical feature calculated by random forest method. The intersection with the red line indicates the point at which the change in importance slowed down. The vertical axis represents the index of importance, and the horizontal axis depicts the chemical features arranged in descending order of importance

Thereafter, we conducted data augmentation using the SMOTE method, which is a pseudo-data augmentation technique introduced by Chawla et al. [32] for this particular library. The SMOTE hyperparameters used in this study are listed in Table 1. Specifically, the number of ROS modulators increased 27-fold from 351 to the same number as the other 9640 compounds, bringing the amount of data to 19,280. The chemical features of the compounds within the library were used as explanatory variables, and 47-chemical features that showed a sharp change in importance of all chemical features yielded by the random forest algorithm were selected (Fig. 2). The ROS production data and 47-chemical features of the library compounds used in this study are presented in Supplementary Information (Additional file 1). Subsequently, DNN training was performed to predict the extent of ROS production [33]. We randomly divided the training data into 90% and the test data into 10% and verified the accuracy of each data. For comparative verification purposes, a random forest algorithm was used. The hyper-parameters of DNN and random forest used in this study are listed in Tables 2, 3, respectively. Using DNN ROS inference model, we developed an in silico screening system to identify ROS modulators. The root mean square error (RMSE) was used as an evaluation index to evaluate this method. The RMSE was calculated using the following equation:

$$RMSE = \sqrt {{\frac{1}{N}\sum\nolimits_{i\; = \;1}^N {(yi - \widehat{yi})^2 } }}$$

**Table 1:** SMOTE hyperparameters

Parameter	Setting value
Sampling strategy	Minority data
Random state	42
The number of nearest neighbors	5

**Table 2:** Hyperparameters of DNN

Parameter	Setting value
Optimizer	Adam
Activation function	relu
Epochs	100
Hidden layers	4
First layer nodes	128
Second layer nodes	64
Third layer nodes	32
Loss function	mse

**Table 3:** Hyperparameters of Random Forest

Parameter	Setting value
The number of trees	100
The maximum depth	None
Random state	0

($yi$; actual value, $\widehat{yi}$; predicted value, $N$; Number of samples)

### In silico screening of seven million commercially available compounds using a ROS inference model

We gathered SDF (Structure-Data File) data on a vast collection of approximately seven million commercially accessible compounds with diverse synthesizable frameworks from the Namiki Shoji Chemical Cupid database. Next, we performed in silico screening of these compound datasets to explore ROS modulator candidates by inferring the magnitude of ROS production.

### Secondary screening through the visualization of chemical space using the GTM method

A GTM is a highly utilized and powerful technique employed for the visualization and reduction of dimensionality in data [34]. It employs a probabilistic nonlinear approach wherein a manifold is trained to accurately represent the data in its original space, which is subsequently transformed into a two-dimensional latent space. In this study, we selected 47-dimensional chemical features of the compounds used in the DNN [35] and employed the GTM method [36] to visually represent their chemical characteristics. The GTM hyperparameters used in this study are listed in Table 4. The resulting map was divided into 64 compartments, and compounds were selected based on their proximity to 351 ROS modulators (Fig. 1) within each compartment and their high ROS inference values.

**Table 4:** Hyperparameters of GTM

Parameter	Setting value
Map size	15, 15
Number of RBFs (basis functions)	5, 5
Standard deviation of RBF	5
λ in the EM algorithm	0.01
Number of iterations	100

### Measurement of ROS production in tobacco BY-2 cultured cells

The tobacco BY-2 (*Nicotiana tabacum* L. ‘Bright Yellow 2’) cultured cells were maintained through weekly dilutions (1/100) of cells in modified Linsmaier and Skoog medium, as described previously [37]. The cell suspension was agitated on a rotary shaker at 95 rpm at 25 °C in the dark.

Cells cultured for 3 d after sub-culturing were used in this experiment. Twenty five milliliters of cells were collected using centrifugation and resuspended in 120 mL of ROS assay buffer (pH 7.0) containing 5 mM MES, 0.5 mM CaCl_2_, 0.5 mM K_2_SO_4_ and (175 mM mannitol. The cells (100 μL) were dispensed into each well of a 96-well white plate (Thermo Fischer scientific, Denmark; No. 236107) using a multichannel pipette with truncated tips. A total of 1 μL of each chemical compound (final concentration 100 μM) was added to the wells. DMSO (1%) was added to the wells of the plate as a solvent control. After 1.5 h of shaking, cryptogein elicitor (final concentration 25 or 50 nM) was added to the wells to induce ROS production. After culturing for 3 h, 1 μL of 20 mM L-012 (FUJIFILM Wako Pure Chemical, Japan) dissolved in ROS assay buffer [38] was added to the wells, and ROS-dependent chemiluminescence was recorded for 1 s using a luminometer (Berthold, Germany).

### Measurement of ROS production in Arabidopsis seedlings

Arabidopsis seedlings (Col-0) grown on 1/2 Murashige and Skoog liquid medium [39] on the 96-well plate for 7 d in continuous light conditions (22 °C) were used in this experiment. Seedlings were placed into each well of a 96-well white plate (Thermo Fischer scientific, Denmark; No. 236107) containing 100 μL ROS assay buffer (pH 7.0; 5 mM MES, 0.5 mM CaCl_2_, 0.5 mM K_2_SO_4_, and 175 mM mannitol). A total of 1 μL of each chemical compound (final concentration 100 μM) was added to the wells. DMSO (1%) was added to the wells of the plate as a solvent control. After chemical treatment for 1 d, 10 μL of 1 mM L-012 dissolved in ROS assay buffer [38] was added to the wells. Subsequently, the flg22 elicitor (final concentration 1 µM) was added to the wells to induce ROS production. ROS-dependent chemiluminescence was recorded every minute for 0.25 s for a duration of 40 min using a luminometer (Berthold, Germany).

### Cell death assay using Evans blue in tobacco BY-2 cultured cells

Cells cultured for 3 d after sub-culturing were used in this experiment. An aliquot of tobacco BY-2 cells was incubated with 0.05% Evans blue (Merck, Germany) for 10 min and washed four times with water to remove any unabsorbed dye. Selective staining of dead cells with Evans blue depends on the extrusion of the dye from living cells via an intact plasma membrane [40]. In this study, > 200 cells in each examination under the bright-field microscope were counted.

### Chemicals

Candidate compounds for ROS modulation were obtained from Namiki Shoji (Tokyo, Japan). L-012, a ROS indicator, was purchased from FUJIFILM Wako Pure Chemical Co. (Osaka, Japan). The flg22-peptide as an elicitor [41], was synthesized and purchased from Eurofins Genomics (Tokyo, Japan).

### Expression and purification of cryptogein elicitor

*Pichia pastoris* (strain GS115) carrying plasmid pLEP3 was used for cryptogein production [26]. Cryptogein was produced as described by O’Donohue et al. [42] and dissolved in distilled water. Cryptogenin concentrations were measured using UV spectroscopy with an extinction coefficient of 8306 M^˗1^ cm^˗1^ at 277 nm [43].

### Statistical analysis

The significance of differences was assessed using the unpaired Student’s *t*-test, with (a) *p* < 0.05, (b) *p* < 0.01, and (c) *p* < 0.005 considered significant.

## Results and discussion

### Construction of ROS inference model

**Fig. 3:**
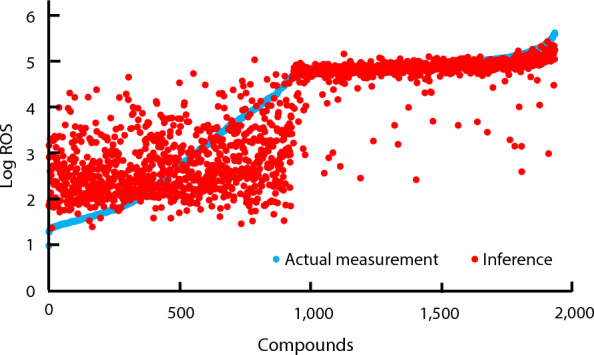
Results of ROS inference and measured values in tobacco BY-2 cultured cells. The vertical axis represents the logarithmic scale of ROS production levels, and the horizontal axis represents the arrangement of compounds in ascending order of ROS production levels

The results of ROS inference using the training data are presented in Fig. 3 and Table 5. After training, the accuracy was verified using the RMSE. DNN showed a higher F-measure compared to random forest, with a precision of over 90%. In contrast, compared to DNN, random forest exhibits a reduced recall rate, indicating that random forest is more likely to overlook candidate compounds. These results suggest that DNN is suitable as a learning model for our screening system.

**Table 5:** Comparison of learning accuracy between the DNN and random forest

	Random forest		DNN	
	Training data	Test data	Training data	Test data
RMSE	0.27	0.73	0.47	0.73
Precision	1	1	0.95	0.93
Recall	0.94	0.86	0.99	0.99
F-measure	0.97	0.92	0.97	0.96

In this validation, ROS modulators, which are potential plant defense activators, were accurately predicted with a high level of precision, exceeding 90% (Fig. 3 and Table 5). The reason for the high accuracy is thought to be that the candidate compounds are amplified by the SMOTE method before training and verification, it is likely that chemical features similar to the candidates will be included in the training data. The high level of accuracy can be attributed to the amplification of candidate compounds through the SMOTE method prior to the training and validation processes, thereby increasing the likelihood of incorporating chemical characteristics similar to the candidate into the training dataset. These results indicate that accurate prediction is possible for a group of compounds with a certain degree of similarity in chemical characteristics to the candidate compounds used for training.

In order to verify the accuracy of compounds with unknown properties, we changed the training data and conducted experiments. Specifically, before the SMOTE work, 10% of the compounds from each of the 351 ROS modulators and 9640 other compounds were randomly removed as verification data, and the DNN model was trained using the rest of data to evaluate the prediction accuracy of the validation data. The prediction precision for compound groups with unknown characteristics that were not part of the training set was approximately 10%, indicating that ROS for compound groups with definitely unknown characteristics that were not part of the training set is hard to predict. Therefore, additional data obtained by expanding the number of compound groups included in the training set may be necessary to accurately predict ROS production for unknown compounds and to effectively search for pesticide compounds.

The model developed in this study demonstrated a remarkably high predictive accuracy for the trained compound group, indicating its efficacy in identifying compounds with specific chemical characteristics (Fig. 3 and Table 5). Recently, a systematic analysis of structure–activity relationships has reported that small structural changes in active compounds, called activity cliffs (ACs), lead to considerable improvements in activity [44]. The ROS screening system is a typical multi-target screening technique because ROS have multiple physiological action points in cells [2], indicating that the discovery of ACs may produce lead compounds with novel MoAs.

Overall, it is inferred that the proposed model enables the selection of a diverse lead compound group with a foundational framework resembling plant defense activators but with distinct MoAs compared to existing chemicals. Consequently, large compound libraries with the potential for structural expansion can be effectively screened using this model, thereby facilitating the identification of novel compound candidates.

### In silico screening of seven million commercially available compounds using the ROS inference model

**Fig. 4:**
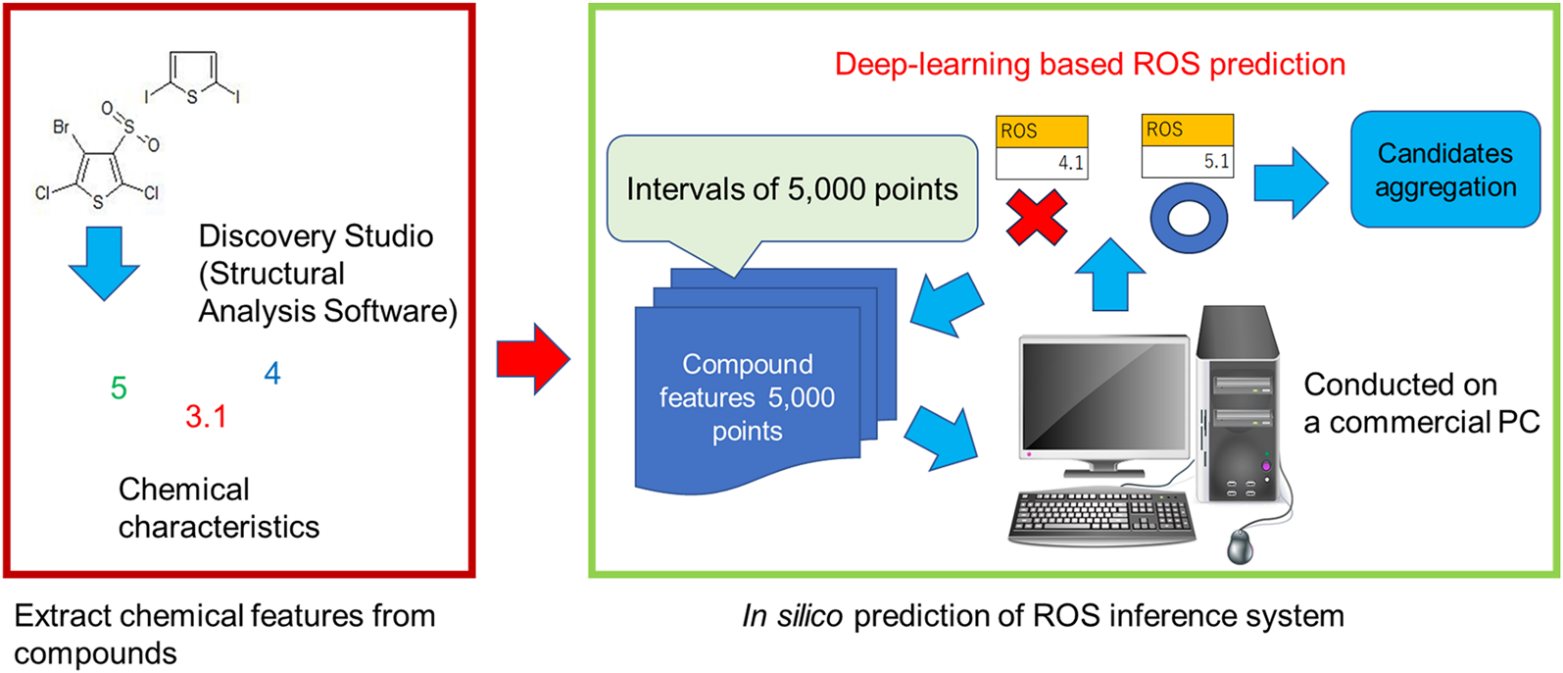
In silico prediction of ROS inference system. In silico prediction of ROS inference system was performed based on deep learning-based prediction of ROS accumulation combined with the chemical properties of the compounds as explanatory variables. The system processing steps were as follows: (1) Chemical feature values for each compound were obtained using Discovery Studio, and the collected data were aggregated at intervals of 5000 points. (2) The ROS inference model was used to determine the ROS production level for each compound. Compounds that exceeded the threshold value (log_10_[4.67]) were identified and consolidated as candidate compounds

Using a ROS inference model, we established an in silico screening system for ROS modulators. A flowchart illustrating the in silico screening system based on the ROS inference model developed in this study is presented in Fig. 4. The system processing steps were as follows: (1) Chemical feature values for each compound were obtained using Discovery Studio, and the collected data were aggregated at intervals of 5000 points. (2) The ROS inference model was used to determine the ROS production level for each compound. Compounds that exceeded the threshold value (log_10_[4.67]) were selected and consolidated as candidate compounds. This system enabled the analysis of one million compounds per day, resulting in 2500 times faster screening speed than that of our previous method [29].

Overall, 8476 compounds were selected from a vast pool of seven million compounds as potential candidates for ROS modulators based on in silico screening using the ROS inference model (Table 6).

**Table 6:** The result of in silico screening system using the ROS inference model combined with the visualization of chemical space of compounds

Screening phase	Number of compounds
Total number of in silico screening	7,003,667
ROS modulator candidates (1st screening)	8476
ROS modulator candidates (2nd screening)	578

### Secondary screening via visualization of chemical space using the GTM method

**Fig. 5:**
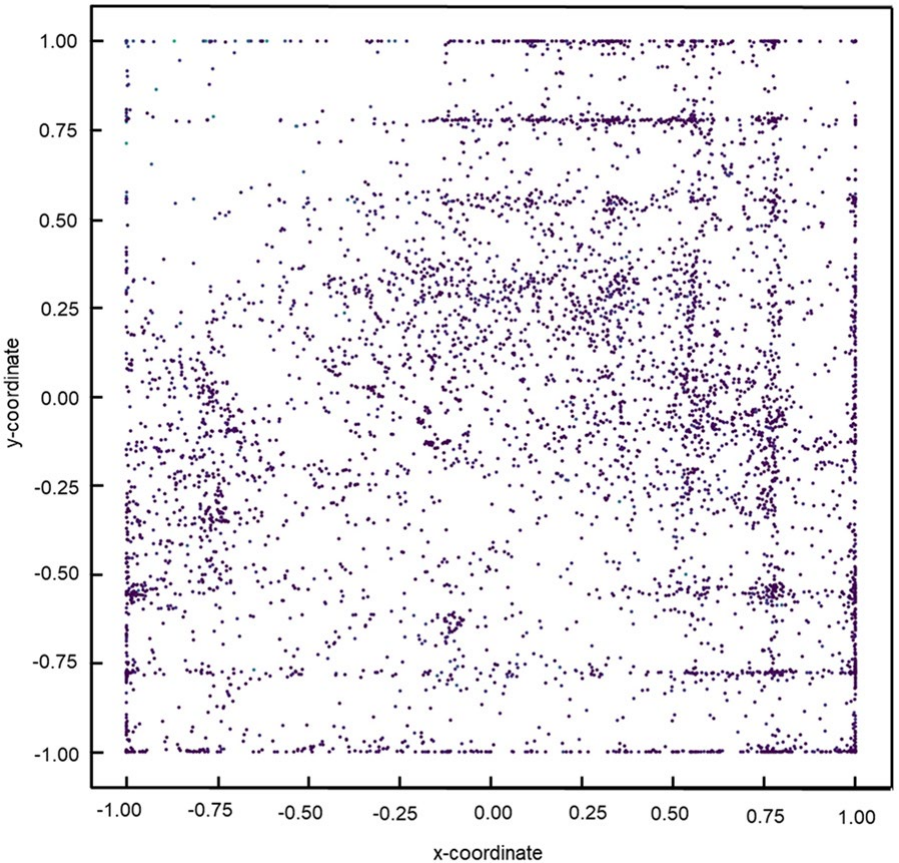
Visualization of chemical space of selected compounds for putative ROS modulators using the GTM method. A chemical feature-based 2D map depicting chemical space visualization was generated using the GTM method for selected compounds from the initial screening. The results obtained from the GTM analysis were divided into 64 categories. The vertical axis represents the chemical spaces ranging from a minimum of − 1.00 to a maximum of 1.00, and the horizontal axis depicts the chemical space spanning from a minimum of − 1.00 to a maximum of 1.00

To effectively and systematically select a diverse group of compounds with distinct chemical characteristics [45], 2D maps of the chemical features of the compounds were generated using the GTM for the secondary selection of candidate compounds [46]. The resulting map was then partitioned into 64 compartments and the compounds were carefully chosen based on their proximity to ROS modulators within the closest distance and their high inference ROS values (Fig. 5). Compounds exhibiting deleterious effects were eliminated from consideration, whereas others identified as potential ROS modulators were subjected to biological assays to evaluate their ROS activity in plant cells. As a result, we screened 578 chemical compounds out of 8476 potential ROS modulators (Table 6).

**Fig. 6:**
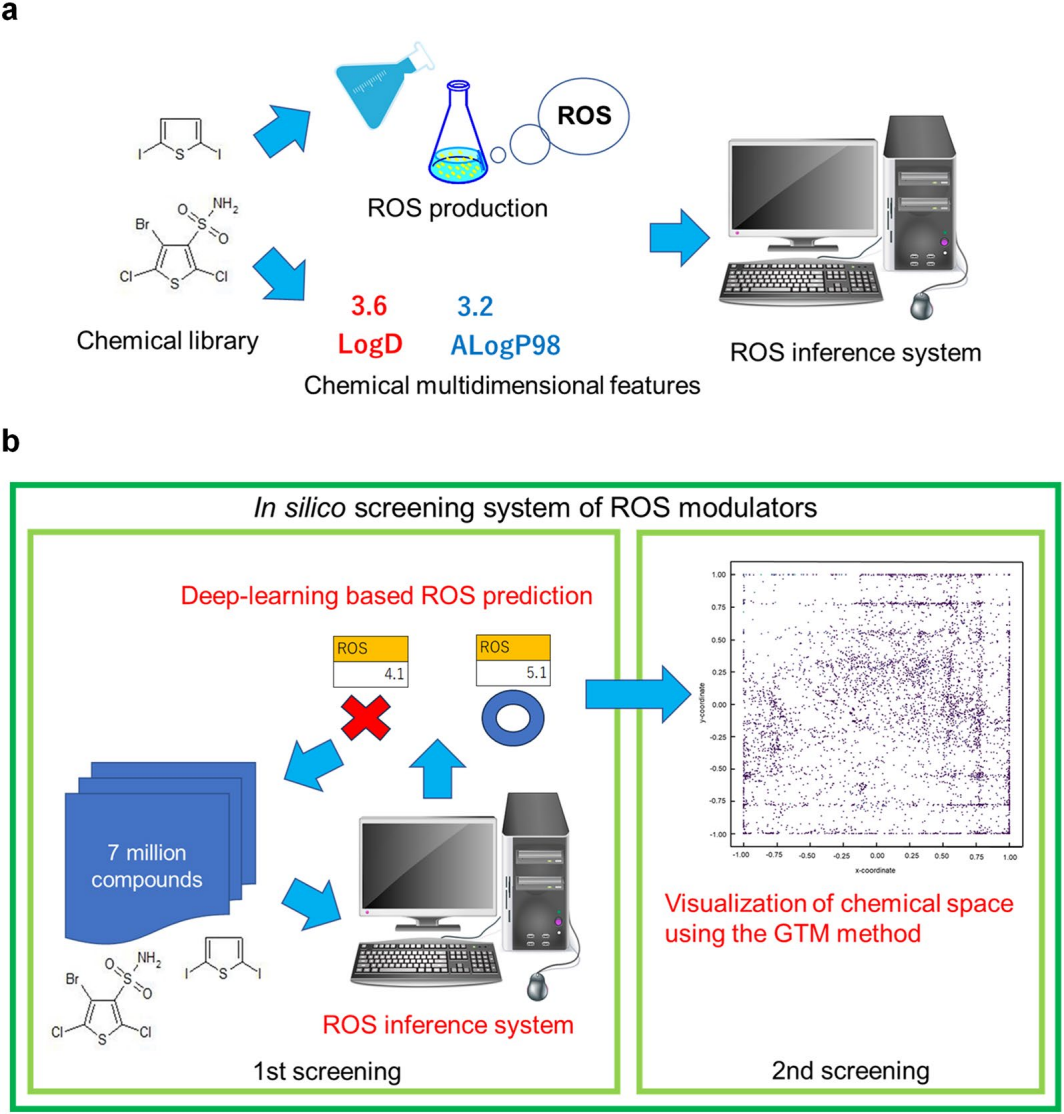
Schematic diagram of the established in silico screening system of ROS modulators. The screening comprised three interconnected phases that allowed the identification of a variety of ROS modulators. The first phase developed a ROS inference system based on DNN learning that combines ROS production data in plant cells and multidimensional chemical features of compounds (**a**). The second phase was in silico first screening of seven million commercially available compounds using the ROS inference model (**b**). The third phase was in silico second screening of selected compounds by visualizing the chemical space using the GTM method (**b**). This system is based on in silico analysis and has advantages in terms of both selection time and cost compared to actual measurements in plant cells

A screening overview and the number of compounds isolated from our chemical screening system based on the ROS inference model, in combination with the visualization of the chemical space of the compounds, are shown in Fig. 6 and Table 6.

### Verification of potential ROS modulators selected using the in silico screening system on the cryptogein-inducible ROS production in tobacco BY-2 cultured cells

**Fig. 7:**
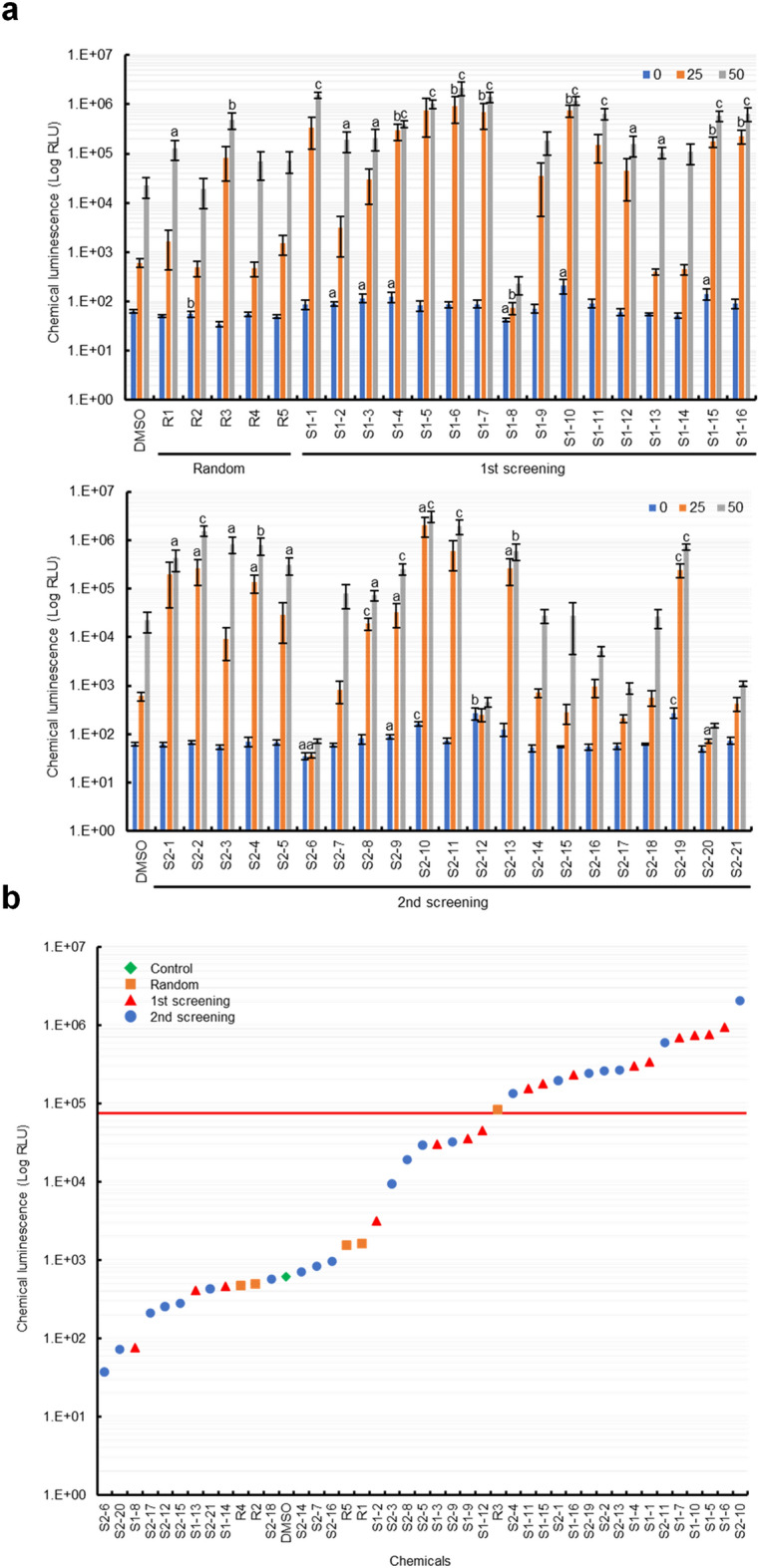
Verification of potential ROS modulators on cryptogein-inducible ROS production in tobacco BY-2 cultured cells. **a** The effect of potential ROS modulators on cryptogein-inducible ROS production in tobacco BY-2 cultured cells. Cultured cells were dispensed into each well of a 96-well white plate, and each compound (final concentration 100 or 50 µM) was added to the indicated wells for 1.5 h. Next, cryptogein (final concentration 25 or 50 nM) was added to the wells and incubated for 3 h. After cryptogein treatment, L-012 was added directly to the wells and ROS-dependent chemiluminescence was recorded using a multi-luminometer. DMSO (1%) was used as a solvent control. Error bars represent SE (n = 3). a < 0.05, b < 0.01, c < 0.005; significantly different from the DMSO control. **b** The vertical axis represents the logarithmic conversion of ROS-dependent chemiluminescence levels in tobacco BY-2 cultured cells triggered by cryptogein (25 nM), and the horizontal axis represents the chemical compounds arranged in ascending order based on their ROS-dependent chemiluminescence levels. Selected criteria (log_10_[4.67]) are shown in red line

To verify whether the potential ROS modulators predicted using the in silico screening system exhibited high ROS activity, we evaluated the effects of the selected compounds on cryptogein-induced ROS production mediated by NADPH oxidase in tobacco BY-2 cultured cells (Fig. 7a). The evaluated compounds were randomly extracted using a random-number generator (https://docs.python.org/3/library/random.html#random.random), and 16 and 21 chemicals were selected from the selected compound groups at each screening stage, respectively. Five control compounds (random compounds) were also randomly selected from 7 million commercially available compounds using a random-number generator. Approximately 56.3% of the potential ROS modulators (9 out of 16 compounds) selected in the first screening stage based on the ROS inference model induced high ROS production triggered by cryptogein in tobacco BY-2 cultured cells compared to that of the DMSO control (Fig. 7b). Moreover, approximately 33.3% of the potential ROS modulators (7 out of 21 compounds) selected in the second screening stage based on the visualization of the chemical space of compounds also induced high ROS production in tobacco BY-2 cultured cells (Fig. 7b). In contrast, most randomly-selected control compounds showed comparable ROS production to that of the DMSO control in tobacco BY-2 cells (Fig. 7), indicating that the compounds selected by the screening system based on the DNN were ROS modulators, with an accuracy of ≥ 43.2% (16 out of 37 compounds). Notably, 351 ROS modulators were selected from the ROS production data out of 9991 chemical compounds of the library (Fig. 1), indicating approximately 3.5% of the hit rate of the ROS modulators obtained from the ROS-measuring system using tobacco BY-2 cultured cells [29].

Overall, our data suggest that DNN-based screening systems can be used to rapidly and effectively identify potential immune signal-inducible ROS modulators with distinct chemical characteristics compared with the actual ROS measurement system.

### Effects of the selected ROS modulators on the flg22-inducible ROS production *in planta*

Plant defense activators are analogs of the defense hormone salicylic acid, which protects plants from pathogens by activating the plant immune system [47–51]. Compared with commonly used pesticides that directly target pathogens, plant defense activators are not pathogen-specific, are not overcome by microbes, and are durable in the field [52]. Several chemical screening procedures have been reported using Arabidopsis seedlings in combination with a promoter reporter system for defense genes as activity markers [53, 54]. However, the compounds identified in these screening studies constitutively activated defense responses and were often associated with arrested growth and reduced yields [55]. Therefore, developing screening systems for novel plant defense activators with novel MoAs is urgently needed.

**Fig. 8:**
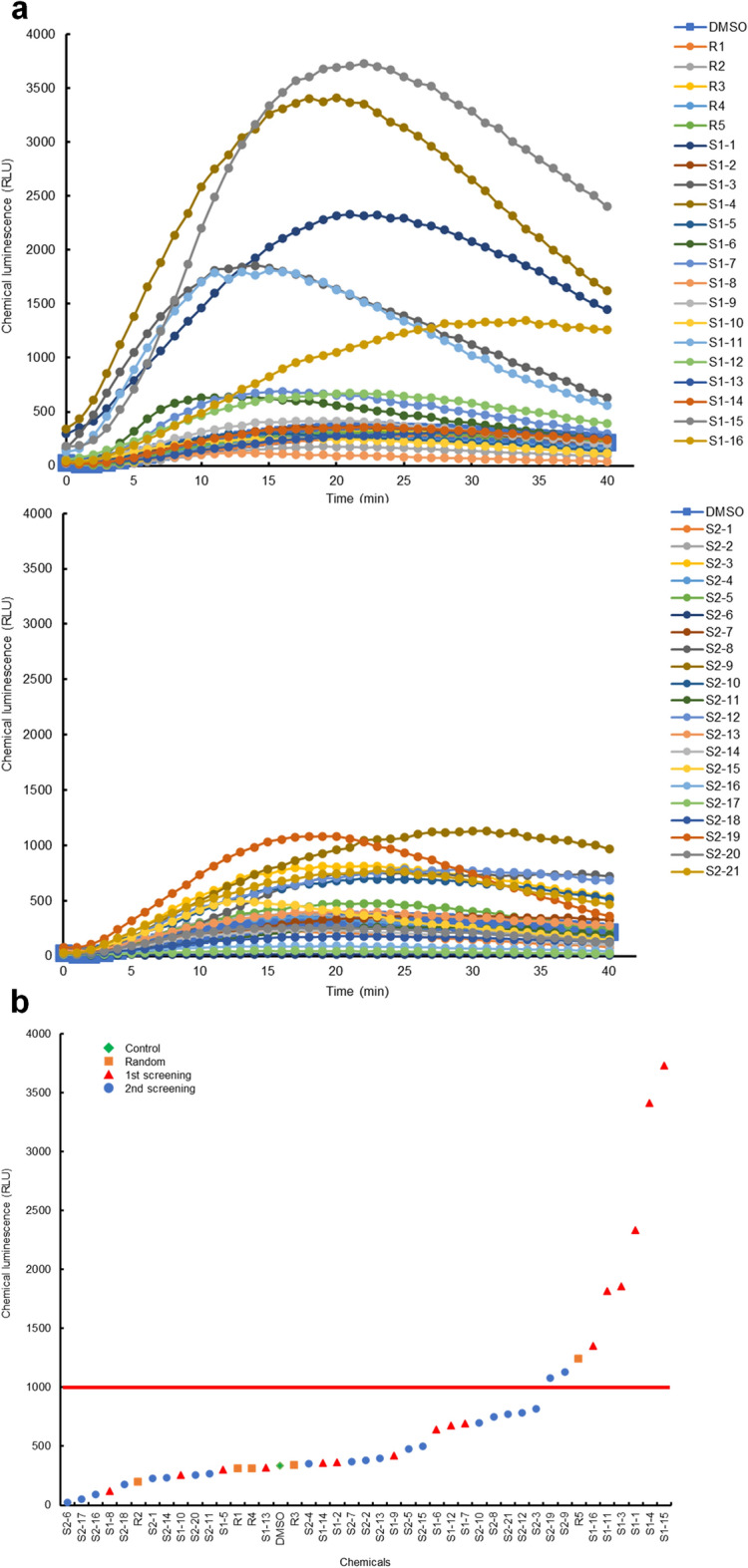
Verification of potential ROS modulators selected on flg22 peptide-inducible ROS production in Arabidopsis seedlings. **a** The effect of potential ROS modulators on flg22 peptide-inducible ROS production in Arabidopsis seedlings. A seedling was placed into each well of a 96-well white plate, and each compound (final concentration 100 or 50 µM) was added to the indicated wells for 24 h. ROS indicator L-012 was added to the wells and then flg22-peptide elicitor (final concentration 1 µM) was added to the wells to induce ROS production. ROS-dependent chemiluminescence was recorded using a multi-luminometer. Selected criteria are shown in red line. **b** The vertical axis represents the peak height of ROS-dependent chemiluminescence levels in Arabidopsis seedlings triggered by flg22 peptide (1 µM), and the horizontal axis represents the chemical compounds arranged in ascending order of their ROS-dependent chemiluminescence levels

We investigated whether the selected compounds could act as ROS modulators in whole plants and in other plant species. As shown in Fig. 8 and Additional file 2, approximately 21.6% of the selected compounds (8 out of 37 compounds) induced high ROS production mediated by NADPH oxidase triggered by the flg22-peptide in Arabidopsis seedlings compared with the DMSO control [56]. In contrast, the control chemicals selected randomly, except for one compound, showed ROS production similar to that of the DMSO control (Fig. 8 and Additional file 2), suggesting that potential ROS modulators could enhance enzymatic ROS production triggered by microbe-associated molecular patterns, signal molecules from microbes, in whole plants and in other plant species.

### Effects of the selected ROS modulators on the cryptogein-inducible PCD in tobacco BY-2 cultured cells

**Fig. 9:**
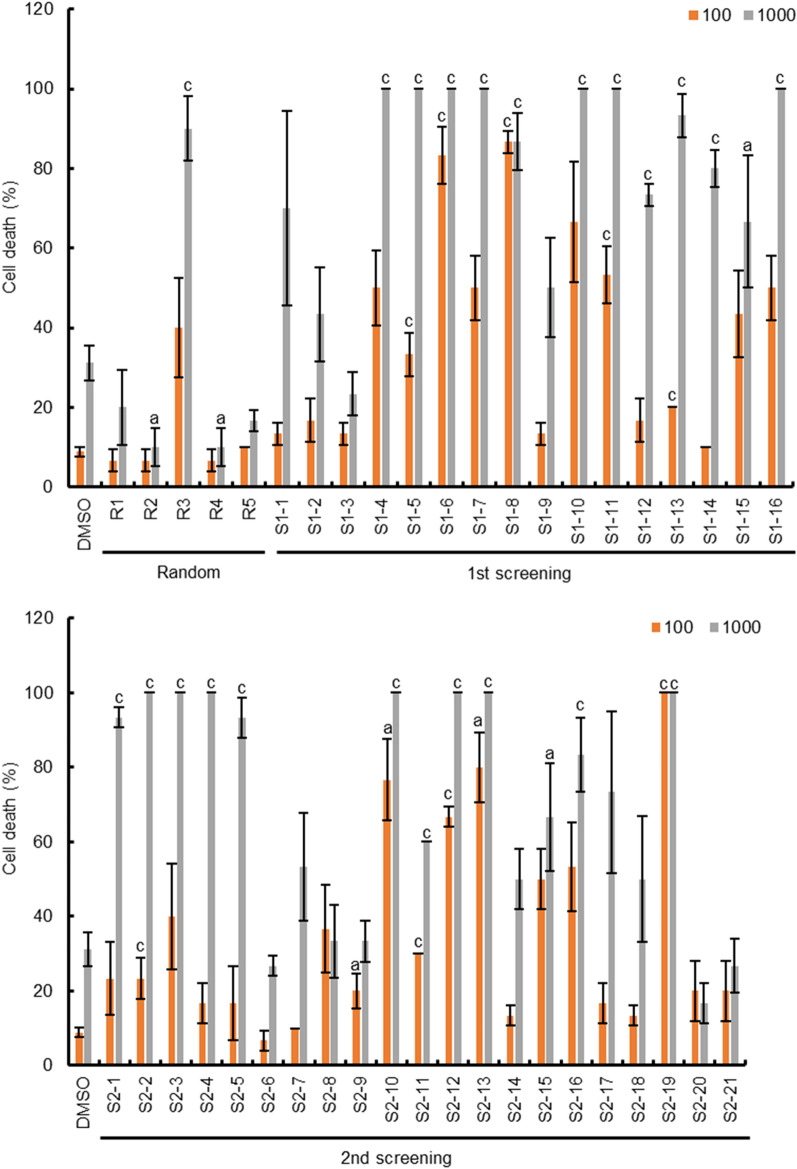
Effects of potential ROS modulators on cryptogein-inducible programmed cell death in tobacco BY-2 cultured cells. Effects of potential ROS modulators on cryptogein-inducible PCD in tobacco BY-2 cultured cells. Cultured cells were dispensed into each well of a 96-well plate, and each compound (final concentration 100 or 50 µM) was added to the indicated wells for 1.5 h. Next, the cryptogein elicitor (final concentration 100 or 1000 nM) was added to the wells and incubated for 24 h. The cells were stained with 1% Evans blue dye (final concentration, 0.05%) for 10 min and washed four times with water. Stained cells were observed under a light microscope. Error bars represent SE (n = 3). a < 0.05, c < 0.005; significantly different from the DMSO control

ROS have direct antimicrobial properties and also serve as signaling molecules that activate further immune responses [57]. When cultured tobacco BY-2 cells are treated with a proteinaceous elicitor derived from a pathogen, cryptogein, immune responses such as persistent ROS production and PCD are induced [26–28]. Therefore, we investigated whether the selected compounds that enhanced ROS production could also induce PCD triggered by cryptogein in tobacco BY-2 cells. As anticipated, almost all ROS modulators selected by the in silico screening system clearly enhanced cryptogein-induced PCD in tobacco BY-2 cultured cells compared with that of the DMSO control (Fig. 9). In contrast, the control chemicals selected randomly, except for one compound, showed PCD similar to that of the DMSO control (Fig. 9). These results suggest that many selected compounds have the potential to be activators of immune responses in plant cells, and our established screening system based on ROS prediction can be used to identify potential plant defense activators.

### Limitations of this study

Our results indicate that accurate ROS prediction by our inference model based on DNN learning is possible for a group of compounds with a certain degree of similarity in chemical characteristics to the candidate compounds used for training (Fig. 3 and Table 5). In contrast, our results show that ROS for compound groups with definitely unknown characteristics that were not part of the training set is hard to predict. Therefore, additional data obtained by expanding the number of compound groups included in the training set may be necessary to accurately predict ROS production for unknown compounds and to effectively search for pesticide compounds. It is expected that efficient learning will be possible by using various compound groups with different chemical characteristics in the dataset.

## Conclusions

Conventional chemical screening of whole plants requires large quantities of chemicals, may be costly, and requires a strictly controlled growth space. ROS are antimicrobial products but also serve as signaling molecules that activate immune responses and have multiple physiological action points in cells, indicating that ROS modulator have the potential to function as a plant defense activator, and the high-throughput screening system of ROS modulator is strongly expected to create lead chemical compounds with novel MoAs. In this study, we established a novel in silico screening system for ROS modulators using deep learning-based prediction of ROS accumulation combined with the chemical properties of the compounds as explanatory variables. The importance of this strategy was demonstrated by identifying candidates for ROS modulators that might function as potential plant defense activators with novel MoAs *in planta* (Figs. 3, 4, 5, 6, 7, 8, 9). This in silico system offers advantages in terms of both time and cost compared with experimental ROS measurements in plant cells, and the discovery of ACs may produce lead compounds with novel MoAs. By employing this system in the prescreening phase of ROS measurement in plant cells, we anticipate enhanced efficiency and reduced pesticide discovery costs. The in silico screening methods for the identification of plant ROS modulators may aid in the development of a variety of plant defense activators that enhance disease tolerance in crops. Additionally, as ROS play multiple beneficial roles as signaling molecules capable of regulating diverse metabolic pathways and gene expression in response to environmental stresses, these findings have the potential to contribute to the development of lead compounds with novel MoAs that confer multiple tolerances against various biotic and abiotic stresses.

### Supplementary Information

**Additional file 1: Table S1.** ROS and chemical features data of the library.

**Additional file 2: Table S2. **The ROS peak height and time of Fig. 8a.

## Data Availability

The learning algorithm codes used during the current study are available on the GitHub address (https://github.com/ma1206ko/in_silico_screening). The datasets used and/or analyzed during the current study are available from the corresponding author on reasonable request.

## References

[1] Umetsu N, Shirai Y (2020). Development of novel pesticides in the 21st century. J Pestic Sci.

[2] Mittler R (2017). ROS are good. Trends Plant Sci.

[3] Kärkönen A, Kuchitsu K (2015). Reactive oxygen species in cell wall metabolism and development in plants. Phytochemistry.

[4] Wrzaczek M, Brosché M, Kangasjärvi J (2013). ROS signaling loops-production, perception, regulation. Curr Opin Plant Biol.

[5] Choudhury FK, Rivero RM, Blumwald E, Mittler R (2017). Reactive oxygen species, abiotic stress and stress combination. Plant J.

[6] Baxter A, Mittler R, Suzuki N (2014). ROS as key players in plant stress signalling. J Exp Bot.

[7] Kurusu T, Kuchitsu K, Tada Y (2015). Plant signaling networks involving Ca^2+^ and Rboh/Nox-mediated ROS production under salinity stress. Front Plant Sci.

[8] Castro B, Citterico M, Kimura S, Stevens DM, Wrzaczek M, Coaker G (2021). Stress-induced reactive oxygen species compartmentalization, perception and signalling. Nat Plants.

[9] Mittler R (2002). Oxidative stress, antioxidants and stress tolerance. Trends Plant Sci.

[10] Gill SS, Tuteja N (2010). Reactive oxygen species and antioxidant machinery in abiotic stress tolerance in crop plants. Plant Physiol Biochem.

[11] Czarnocka W, Karpiński S (2018). Friend or foe? Reactive oxygen species production, scavenging and signaling in plant response to environmental stresses. Free Radic Biol Med.

[12] Caverzan A, Piasecki C, Chavarria G, Stewart CN, Vargas L (2019). Defenses against ROS in crops and weeds: The effects of interference and herbicides. Int J Mol Sci.

[13] Durner J, Wendehenne D, Klessig DF (1998). Defense gene induction in tobacco by nitric oxide, cyclic GMP, and cyclic ADP-ribose. Proc Natl Acad Sci USA.

[14] Torres MA, Dangl JL, Jones JDG (2002). Arabidopsis gp91phox homologues AtrbohD and AtrbohF are required for accumulation of reactive oxygen intermediates in the plant defense response. Proc Natl Acad Sci USA.

[15] Dempsey DA, Klessig DF (1994). Salicylic acid, active oxygen species and systemic acquired resistance in plants. Trends Cell Biol.

[16] Torres MA, Jones JDG, Dangl JL (2006). Reactive oxygen species signaling in response to pathogens. Plant Physiol.

[17] Kadota Y, Shirasu K, Zipfel C (2015). Regulation of the NADPH oxidase RBOHD during plant immunity. Plant Cell Physiol.

[18] Qi J, Wang J, Gong Z, Zhou JM (2017). Apoplastic ROS signaling in plant immunity. Curr Opin Plant Biol.

[19] Schieber M, Chandel NS (2014). ROS function in redox signaling and oxidative stress. Curr Biol.

[20] Wei Y, Kenyon C (2016). Roles for ROS and hydrogen sulfide in the longevity response to germline loss in *Caenorhabditis elegans*. Proc Natl Acad Sci USA.

[21] Jalal A, Oliveira Junior JC, Ribeiro JS, Fernandes GC, Mariano GG, Trindade VDR (2021). Hormesis in plants: physiological and biochemical responses. Ecotoxicol Environ Saf.

[22] Iwata M, Suzuki Y, Watanabe T, Mase S, Sekizawa Y (1980). Effect of probenazole on the activities of enzymes related to the resistant reaction in rice plant. Japanese Journal of&nbsp; Phytopathology.

[23] Yoshioka K, Nakashita H, Klessig DF, Yamaguchi I (2001). Probenazole induces systemic acquired resistance in Arabidopsis with a novel type of action. Plant J.

[24] Nakashita H, Yoshioka K, Yasuda M, Nitta T, Arai Y, Yoshida S (2002). Probenazole induces systemic acquired resistance in tobacco through salicylic acid accumulation. Physiol Mol Plant Pathol.

[25] Yasuda M, Kusajima M, Nakajima M, Akutsu K, Kudo T, Yoshida S (2006). Thiadiazole carboxylic acid moiety of tiadinil, SV-03, induces systemic acquired resistance in tobacco without salicylic acid accumulation. J Pestic Sci.

[26] Kadota Y, Goh T, Tomatsu H, Tamauchi R, Higashi K, Muto S (2004). Cryptogein-induced initial events in tobacco BY-2 cells: Pharmacological characterization of molecular relationship among cytosolic Ca^2+^ transients, anion efflux and production of reactive oxygen species. Plant Cell Physiol.

[27] Kadota Y, Watanabe T, Fujii S, Higashi K, Sano T, Nagata T (2004). Crosstalk between elicitor-induced cell death and cell cycle regulation in tobacco BY-2 cells. Plant J.

[28] Kurokawa M, Nakano M, Kitahata N, Kuchitsu K, Furuya T (2021). An efficient direct screening system for microorganisms that activate plant immune responses based on plant-microbe interactions using cultured plant cells. Sci Rep.

[29] Kuchitsu K, Kurusu T. Method of screening for plant defense activators, plant defense activators, and method of enhancing immune response. Patent: WO2012029539. 2011.

[30] Chen H, Engkvist O, Wang Y, Olivecrona M, Blaschke T (2018). The rise of deep learning in drug discovery. Drug Discov Today.

[31] Neves BJ, Braga RC, Melo-Filho CC, Moreira-Filho JT, Muratov EN, Andrade CH (2018). QSAR-based virtual screening: advances and applications in drug discovery. Front Pharmacol.

[32] Chawla NV, Bowyer K, Hall LO, Kegelmeyer WP (2002). SMOTE: synthetic minority over-sampling technique. J Artif Intell Res.

[33] Krizhevsky A, Sutskever I, Hinton GE (2017). ImageNet classification with deep convolutional neural networks. Commun ACM.

[34] Kireeva N, Baskin II, Gaspar HA, Horvath D, Marcou G, Varnek A (2012). Generative topographic mapping (GTM): Universal tool for data visualization, structure-activity modeling and dataset comparison. Mol Inform.

[35] Horvath D, Marcou G, Varnek A (2019). Generative topographic mapping in drug design. Drug Discov Today Technol.

[36] Bishop CM, Svensén M, Williams CKI (1998). GTM: The generative topographic mapping. Neural Comput.

[37] Nagata T, Nemoto Y, Hasezawa S (1992). Tobacco BY-2 cell-line as the "Hela" cell in the cell biology of higher plants. Int Rev Cytol.

[38] Nishida A, Misaki Y, Kuruta H, Takashima S (1994). Developmental expression of copper, zinc-superoxide dismutase in human brain by chemiluminescence. Brain Dev.

[39] Murashige T, Skoog F (1962). A revised medium for rapid growth and bioassays with tobacco tissue cultures. Physiol Plant.

[40] Turner JG, Novacky A (1974). The quantitative relation between plant and bacterial cells involved in the hypersensitive reaction. Phytopathology.

[41] Denoux C, Galletti R, Mammarella N, Gopalan S, Werck D, De Lorenzo G (2008). Activation of defense response pathways by OGs and Flg22 elicitors in Arabidopsis seedlings. Mol Plant.

[42] O’Donohue MJ, Boissy G, Huet JC, Nespoulous C, Brunie S, Pernollet JC (1996). Overexpression in *pichia pastoris* and crystallization of an elicitor protein secreted by the phytopathogenic fungus *Phytophthora cryptogea*. Protein Expr Purif.

[43] O’Donohue MJ, Gousseau H, Huet JC, Tepfer D, Pernollet JC (1995). Chemical synthesis, expression and mutagenesis of a gene encoding beta-cryptogein, an elicitin produced by *Phytophthora cryptogea*. Plant Mol Biol.

[44] Asawa Y, Yoshimori A, Bajorath J, Nakamura H (2020). Prediction of an MMP-1 inhibitor activity cliff using the SAR matrix approach and its experimental validation. Sci Rep.

[45] Korn M, Ehrt C, Ruggiu F, Gastreich M, Rarey M (2023). Navigating large chemical spaces in early-phase drug discovery. Curr Opin Struct Biol.

[46] Sidorov P, Viira B, Davioud-Charvet E, Maran U, Marcou G, Horvath D (2017). QSAR modeling and chemical space analysis of antimalarial compounds. J Comput Aided Mol Des.

[47] Görlach J, Volrath S, Knauf-Beiter G, Hengy G, Beckhove U, Kogel KH (1996). Benzothiadiazole, a novel class of inducers of systemic acquired resistance, activates gene expression and disease resistance in wheat. Plant Cell.

[48] Lawton KA, Friedrich L, Hunt M, Weymann K, Delaney T, Kessmann H (1996). Benzothiadiazole induces disease resistance in Arabidopsis by activation of the systemic acquired resistance signal transduction pathway. Plant J.

[49] Noutoshi Y, Okazaki M, Kida T, Nishina Y, Morishita Y, Ogawa T (2012). Novel plant immune-priming compounds identified via high-throughput chemical screening target salicylic acid glucosyltransferases in Arabidopsis. Plant Cell.

[50] Kumar D (2014). Salicylic acid signaling in disease resistance. Plant Sci.

[51] Takatsuji H (2014). Development of disease-resistant rice using regulatory components of induced disease resistance. Front Plant Sci.

[52] Watanabe T, Igarashi H, Matsumoto K, Seki S, Mase S, Sekizawa Y (1977). The characteristics of probenazole (Oryzemate) for the control of rice blast. J Pestic Sci.

[53] Serrano M, Robatzek S, Torres M, Kombrink E, Somssich IE, Robinson M (2007). Chemical interference of pathogen-associated molecular pattern-triggered immune responses in Arabidopsis reveals a potential role for fatty-acid synthase type II complex-derived lipid signals. J Biol Chem.

[54] Knoth C, Salus MS, Girke T, Eulgem T (2009). The synthetic elicitor 3,5-dichloroanthranilic acid induces NPR1-dependent and NPR1-independent mechanisms of disease resistance in Arabidopsis. Plant Physiol.

[55] He Z, Webster S, He SY (2022). Growth-defense trade-offs in plants. Curr Biol.

[56] Kadota Y, Sklenar J, Derbyshire P, Stransfeld L, Asai S, Ntoukakis V (2014). Direct regulation of the NADPH oxidase RBOHD by the PRR-associated kinase BIK1 during plant immunity. Mol Cell.

[57] Ding LN, Li YT, Wu YZ, Li T, Geng R, Cao J (2022). Plant disease resistance-related signaling pathways: recent progress and future prospects. Int J Mol Sci.

